# Integrating Multi‐Omics Data Using Machine Learning to Explore New Therapeutic Targets for Acute Kidney Injury

**DOI:** 10.1111/jcmm.70801

**Published:** 2025-08-27

**Authors:** Qiming Gong, Yakun Wang, Fahui Liu, Tingting Zhou, Mengqin Tu, Junle Li, Wei Huang, Xu Lin, Wenjuan Sun

**Affiliations:** ^1^ Department of Nephrology Affiliated Hospital of Youjiang Medical University for Nationalities Baise China; ^2^ Key Laboratory of Medical Research Basic Guarantee for Immune‐Related Diseases Research of Guangxi Baise China; ^3^ Department of Nephrology Xin Hua Hospital Affiliated to Shanghai Jiao Tong University School of Medicine Shanghai China; ^4^ Department of Nephrology Qingpu Branch of Zhongshan Hospital Affiliated to Fudan University Shanghai China; ^5^ Xiamen Cell Therapy Research Center, the First Affiliated Hospital of Xiamen University, School of Medicine Xiamen University Xiamen China; ^6^ Department of Endocrinology and Metabolism The Affiliated Hospital of Southwest Medical University Luzhou Sichuan China; ^7^ Department of Nephrology Shanghai Sixth People's Hospital Shanghai China

**Keywords:** ADAMTS1, apoptosis, renal ischemia–reperfusion injury, single‐cell, troglitazone

## Abstract

Renal ischemia–reperfusion (I/R) injury is an unavoidable complication associated with renal transplantation, and currently, there are no targeted therapeutic interventions. The objective of this study was to explore the molecular mechanisms that contribute to I/R‐induced acute kidney injury (I/R‐AKI) and to discover potential targets for effective renal safeguarding. Bioinformatics techniques were employed to analyse critical genes regulating I/R‐AKI at the single‐cell level and to develop diagnostic models. Additionally, key pharmacological agents that inhibit the expression of target genes were identified for subsequent experimental validation. Pathological changes in the kidneys of I/R mice and patients with AKI were observed using immunofluorescence, western blotting, immunohistochemistry and transmission electron microscopy. We developed and validated a robust diagnostic model for I/R‐AKI. The results suggest that ADAMTS1 acts as a promoter of renal I/R‐AKI. In I/R‐AKI, ADAMTS1 was significantly upregulated in renal tubular epithelial cells. Furthermore, apoptosis mediated by the mitochondrial pathway was a critical factor in the progression of renal I/R injury. In mouse models of I/R‐AKI, the inhibition of ADAMTS1 with troglitazone significantly reduced both functional and histological damage. The diagnostic model can serve as a valuable instrument for diagnosing I/R‐AKI. Furthermore, troglitazone can significantly contribute to managing I/R‐AKI by inhibiting the expression of ADAMTS1. This study provides critical insights that may inform future research on therapeutic targets for renal ischaemia–reperfusion injury.

## 1 Introduction

Acute kidney injury (AKI) is a prevalent clinical emergency marked by a rapid decrease in kidney function, including both structural and functional impairment. It is frequently associated with diverse risk factors, including renal ischemia–reperfusion, sepsis, concurrent use of nephrotoxic drugs, exposure to contrast agents and hypotension. Its high incidence and mortality rates characterise AKI and are a primary risk factor for chronic kidney disease (CKD) [1]. Currently, effective clinical treatments for AKI are limited. During AKI progression, various cell types play pivotal roles in the renal microenvironment, including renal tubular epithelial cells, endothelial cells, immune cells and fibroblasts. Furthermore, mitochondrial damage in different cells is now recognised as a critical mediator in the transition from AKI to CKD. However, mitochondrial damage in AKI involves multiple mechanisms, including apoptosis [2], autophagy [3] and ferroptosis [4], each regulated by distinct genes. The intricate interplay between cells and molecular regulation poses a significant challenge in identifying effective therapeutic targets for AKI.

Cell death in AKI can occur through various mechanisms, including apoptosis, pyroptosis, autophagy and ferroptosis. Studies have reported that necroptotic apoptosis in myeloid cells plays an important role in AKI. The production of inflammatory factors, including TNF‐α, is reduced by inhibiting EP3 in myeloid cells. This alleviates necroptotic apoptosis and partially shifts the cell death mode from necroptotic apoptosis to relatively milder apoptosis by suppressing the upregulation of caspase‐8 through the IL‐6/JAK2/STAT3 pathway, thereby reducing AKI [5]. Additional studies have suggested that pyroptosis mediated by caspase‐3/GSDME and inflammation in renal tubular epithelial cells contributes to AKI [6]. Moreover, studies have reported the impact of autophagy on the progression of AKI. The upregulation of autophagy by αKlotho can alleviate ischaemic injury and delay the progression of AKI to CKD [7]. Studies suggest that inhibiting various forms of cell death in the AKI microenvironment can be an effective method to alleviate AKI, including ferroptosis inhibitors, which have demonstrated efficacy in improving fibrosis progression in mouse models of AKI [8]. Moreover, studies have found that directly blocking apoptosis can reduce apoptosis in renal tubular epithelial cells. However, the occurrence and progression of AKI may be exacerbated by the potential for other forms of injury or cell death if upstream‐inducing factors are not eliminated [9]. Consequently, the studies on these drugs are currently limited to the experimental stage, and their clinical application remains restricted.

In our study, we integrated single‐cell transcriptomics with conventional transcriptome data to develop an effective model for enhancing the accuracy of AKI diagnosis. This model facilitated a deeper understanding of the complex pathophysiology of AKI and helped identify a set of key target genes. Based on this, we identified a critical gene, ADAMTS1, playing a significant role in AKI pathophysiology. In a mouse model, we demonstrated the expression of ADAMTS1 in AKI and identified effective drugs targeting ADAMTS1 to alleviate AKI symptoms. These findings provide new perspectives for AKI treatment, suggesting the potential of targeted gene‐based drug therapy in AKI management. This approach advances our understanding of the pathophysiology of AKI but also provides a foundation for future clinical interventions.

## 2 Materials and Methods

### 2.1 Data Acquisition and Processing

Single‐cell data from two cases of AKI (GSE174220) and three samples of normal kidney tissue (GSE131685) were downloaded from the Gene Expression Omnibus (GEO) database. The percentages of mitochondrial, ribosomal and erythroid genes were calculated. Cells expressing more than 300 genes, with less than 50% mitochondrial gene expression and < 1% erythroid gene proportion, were selected. Subsequently, the data were merged for normalisation. The top 2000 highly variable genes were identified using the FindVariableFeature function, and all genes were scaled using the ScaleData function. Dimensionality reduction was performed on the selected top 2000 variable genes using the RunPCA function. The batch correction was then conducted using the harmony algorithm. Cell clustering was achieved using the ‘FindNeighbors’ and ‘FindClusters’ functions (resolution = 0.8). Cell types were annotated and visualised based on the original literature and Cellmarker 2.0. Transcriptome data for AKI were obtained for GSE43974 (training set) and GSE126805 (test set) from the GEO database. Differential genes between the normal and AKI groups were calculated using the limma package, with selection criteria of *p*‐value < 0.05 and Log2 FC > log2 (1.1).

### 2.2 Acquisition of Mitochondria‐Programmed Cell Death Crosstalk Genes

Key regulatory genes of 18 different types of cell death were obtained from the previous study, including 580 apoptosis‐related genes, 367 autophagy‐related genes, 7 alkaliptosis‐related genes, 338 anoikis‐related genes, 19 cuproptosis‐related genes, 15 enteric cell death‐related genes, 87 ferroptosis‐related genes, 34 immunogenic cell death‐related genes, 220 lysosome‐dependent cell death‐related genes, 101 necroptosis‐related genes, 8 netotic cell death‐related genes, 24 NETosis‐related genes, 5 oxeiptosis‐related genes, 52 pyroptosis‐related genes, 9 parthanatos‐related genes and 66 paraptosis‐related genes. In addition, there were 8 Methuosis genes and 23 Entosis genes, totaling 1964 genes associated with programmed cell death. After removing 416 duplicate genes, our analysis included a cluster of 1548 unique genes associated with programmed cell death. Furthermore, we obtained 1136 mitochondria‐related genes from MitoCarta 3.0.

The process of obtaining the mitochondrial‐cell death crosstalk genes is as follows: First, we extracted expression matrices of programmed cell death‐related genes and mitochondrial‐related genes from GSE43974. Pearson's correlation analysis was performed on each programmed cell death‐related gene in the dataset and all mitochondrial genes, retaining genes that met the *p* < 0.05 and *r* > 0.4 criteria. The identified genes were subsequently intersected with the differentially expressed genes obtained from the differential analysis of GSE43974. Finally, 24 genes were identified as key crosstalk genes involved in mitochondria–programmed cell death (mtPCD) in AKI.

### 2.3 Bioinformatics Analysis of Single‐Cell Data

We utilised the R package CellChat (V1.6.0) for cell communication analysis, employing single‐cell data along with our cell categorisation. The CellChat analysis involved using the built‐in CellChatDB human as a reference to analyse the interactions between cells and determine the relationships of 32 pathways among cells. The SCENIC package based on R3.1.4 was used to analyse the regulatory networks of transcription factors in various cell subgroups. During the SCENIC analysis, gene‐motif rankings from the RcisTarget database's hg19‐tss‐centered‐10 kb were utilised for enrichment analysis to determine regulatory networks of the transcription start site with genes in specific subgroups. Visualisation was achieved using the pheatmap package. Gene ontology (GO) annotation analysis and Kyoto Encyclopedia of Genes and Genomes (KEGG) pathway enrichment analysis of differentially expressed genes were performed using the clusterProfiler package for functional enrichment analysis (GO/KEGG/GSVA). Enrichment terms with a *p* < 0.05 were considered to be significantly enriched. Besides, high dimensional weighted gene co‐expression network analysis (hdWGCNA) was employed to determine and identify gene modules significantly correlated with the key subgroup of renal tubular epithelial cells (mtPCD‐TEC).

### 2.4 Construction and Validation of the Diagnostic Model

To develop a model with high accuracy and stability, we integrated 10 machine learning algorithms and 108 algorithm combinations. The comprehensive set of algorithms included Random Survival Forest (RSF), Elastic Net (Enet), Least Absolute Shrinkage and Selection Operator (LASSO), Ridge, Stepwise Cox, CoxBoost, Partial Least Squares Cox Regression (plsRcox), Supervised Principal Components (SuperPC), Generalised Boosted Regression Model (GBM) and Survival Support Vector Machine (survival‐SVM). The signature was generated as follows: (1) Intersection of the hdWGCNA module genes with the differentially expressed genes in the training set was performed to filter key module genes. (2) Next, 108 algorithm combinations were applied to these key genes to fit predictive models based on the Leave‐One‐Out Cross‐Validation (LOOCV) framework. (3) All models were evaluated on the validation dataset. (4) Harrell's concordance index (C‐index) was calculated across all validation datasets for each model. The average C‐index was also calculated and utilised to rank and determine the optimal combination of models.

### 2.5 Assessment of Immune Infiltration Levels

To evaluate the immune cell infiltration levels in AKI samples, three different algorithms were used to conduct a comprehensive analysis. First, the ESTIMATE algorithm was used to calculate StromalScore and ImmuneScore for each sample, which facilitated the estimation of the proportion of non‐tumour cells within the tumour microenvironment. Subsequently, transcriptional data of AKI was analysed using the MCP‐counter method, enabling robust quantification of eight immune cell populations and two stromal cell populations in heterogeneous tissues. Furthermore, the single sample Gene Set Enrichment Analysis (ssGSEA) method was employed to determine the infiltration levels of an additional 28 distinct immune cell types.

### 2.6 Drug Prediction and Molecular Docking

To uncover potential therapeutic agents, we performed an in‐depth analysis using the Comparative Toxicogenomics Database (CTD) (https://ctdbase.org/). By determining the association between hub genes and drugs, we identified key compounds that exert a significant downregulatory effect on hub gene expression. To further elucidate the structural characteristics of these promising drugs, we acquired their chemical structures from PubChem (https://pubchem.ncbi.nlm.nih.gov/), while essential gene structural information was retrieved from the Protein Data Bank (PDB) (https://www.rcsb.org/). Subsequently, molecular docking simulations were performed using the Autodock software to investigate the intricate interactions between the selected drugs and their respective target genes. Pymol was used to visualise these complex interactions.

### 2.7 Human Subjects

Kidney tissue samples were collected from patients diagnosed with AKI through renal biopsy at the Department of Pathology, The Sixth People's Hospital of Shanghai, affiliated with Shanghai Jiao Tong University (Shanghai, China). In addition, control samples were obtained from normal kidney tissues of patients who underwent nephrectomy due to renal carcinoma, ensuring that these patients did not have any other renal diseases. All procedures conducted in this study received approval from the institutional review committee of The Sixth People's Hospital of Shanghai, and each patient provided written informed consent prior to participation in the research.

### 2.8 Animal Models

C57BL/6 mice, a common model organism in biomedical research, were sourced from Genechem Animal Co. Ltd. (Registration No. SYXK 2015‐0008). These mice were housed in a specific pathogen‐free (SPF) facility at Youjiang Medical University for Nationalities, where they were kept under meticulously controlled environmental conditions. The mice were subjected to a regulated 12‐h light/dark cycle, experienced a consistent humidity level of 50%, and were maintained within a temperature range of 20°C–24°C. Additionally, the mice had unlimited access to sterile water and food, ensuring their well‐being and minimising external variables that could affect the study's outcomes. For the purposes of this research, the mice were randomly assigned to one of three distinct groups: the control group, the ischemia/reperfusion (I/R) group, and the treated with Troglitazone (I/*R* + TGZ) group, which allows for a comprehensive analysis of the experimental variables.

The control group was only subjected to sham surgery without ischaemia. I/R group: a laparotomy was performed to expose the abdominal cavity. Mice were anaesthetised with 2% isoflurane. Afterward, the renal pedicle was clamped with microvascular clips for 30 min to induce renal ischaemia. Body temperature was maintained. All mice were euthanised after 24 and 48 h of reperfusion. I/*R* + TGZ group: mice were orally administered Troglitazone (TGZ; 200 mg/kg) daily for five weeks [10] and subjected to ischaemia–reperfusion treatment.

### 2.9 Renal Function Assessment

In this study, the levels of blood urea nitrogen (BUN) and serum creatinine (Scr) in mice were assessed in accordance with the manufacturer's established guidelines. The assessment was conducted using specific kits provided by the Jiancheng Bioengineering Institute (Nanjing, China; kit numbers C013‐2‐1 and C011‐2‐1, respectively).

### 2.10 Western Blotting

The total proteins in mice kidneys were measured using the specific anti‐ADAMTS1 (1:1000, DF9168, Affinity Biosciences), anti‐TNF‐α (1:750, AF7014, Affinity Biosciences), anti‐Bax (1:1000, AF0120, Affinity Biosciences), anti‐Bcl‐2 (1:1000, AF6139, Affinity Biosciences), anti‐PCG1‐α (1:1000, Af5395, Affinity Biosciences), anti‐UCP2 (1:750, DF8626, Affinity Biosciences), and anti‐Cyt‐c (1:1000, AF0146, Affinity Biosciences) primary antibody followed by IRDye 800rd goat anti‐rabbit (1:10,000, SA535571, Invitrogen, USA) secondary antibody. Finally, proteins were visualised by the Licor Odyssey scanner (Licor Bioscience, Lincoln, USA).

### 2.11 Histopathology and Immunohistochemistry

Kidney tissue sections (4 μm) were fixed in 4% paraformaldehyde embedded in paraffin. After deparaffinisation and rehydration, the sections were stained with haematoxylin & eosin (HE) and periodic acid‐Schiff (PAS). Immunohistochemistry (IHC) was performed on the kidney tissue sections (4 μm) using anti‐TNF‐α, anti‐F4‐80, anti‐NGAL, or anti‐Kim‐1 primary antibody (all from Affinity Biosciences, OH, USA; 1:200) and incubated with horseradish peroxidase‐conjugated secondary antibody (Dako, Glostrup, Denmark) at a 1:200 dilution at room temperature for 1 h. The images were visualised using a Nikon microscope imaging system (Nikon, Tokyo, Japan).

### 2.12 Immunofluorescence

The immunofluorescence (IF) experiments were performed using the previously described protocol [11]. Kidney frozen sections (3 μm) were fixed with 4% paraformaldehyde and subjected to IF staining. Tissue sections were immunostained overnight at 4°C with primary antibodies against ADAMTS1, AQP1, F4/80 and Bax. Subsequently, the sections were incubated with goat anti‐rabbit antibodies for 1 h. Tissue sections were imaged using a fluorescence microscope (Olympus, Tokyo, Japan).

### 2.13 Transmission Electron Microscopy

Fresh kidney tissues (1 mm^3^) were fixed in transmission electron microscopy (TEM) fixative (P1126, Servicebio, Beijing, China) at 4°C for 2 h. The tissues were sectioned into ultrathin slices (60–80 nm) and incubated with uranyl acetate and lead citrate. The specimens were then examined using TEM (HT7700, HITACHI, Tokyo, Japan).

### 2.14 TUNEL Assay

The ApopTag Plus Peroxidase in Situ Apoptosis Detection Kit (Chemicon International, Temecula, CA, USA) was used to detect apoptosis in mouse kidney cells following the manufacturer's instructions. Kidney sections were examined using optical and electron microscopy at 40× magnification.

### 2.15 Statistical Analysis

Data were presented in the form of mean ± standard deviations, which provide a comprehensive overview of the central tendency and variability within the data set. The statistical analysis was conducted using GraphPad Prism 9. To evaluate differences between the various groups studied, we employed both the Student's two‐tailed unpaired *t*‐test and Tukey's test. For situations that required multiple comparisons, Tukey's test was applied to ensure accurate assessments of the statistical significance of the differences observed. A *p*‐value of less than 0.05 was established as the threshold for statistical significance.

## 3 Results

### 3.1 Single‐Cell Landscape of AKI; the Activity Score of mtPCD in AKI

**FIGURE 1:**
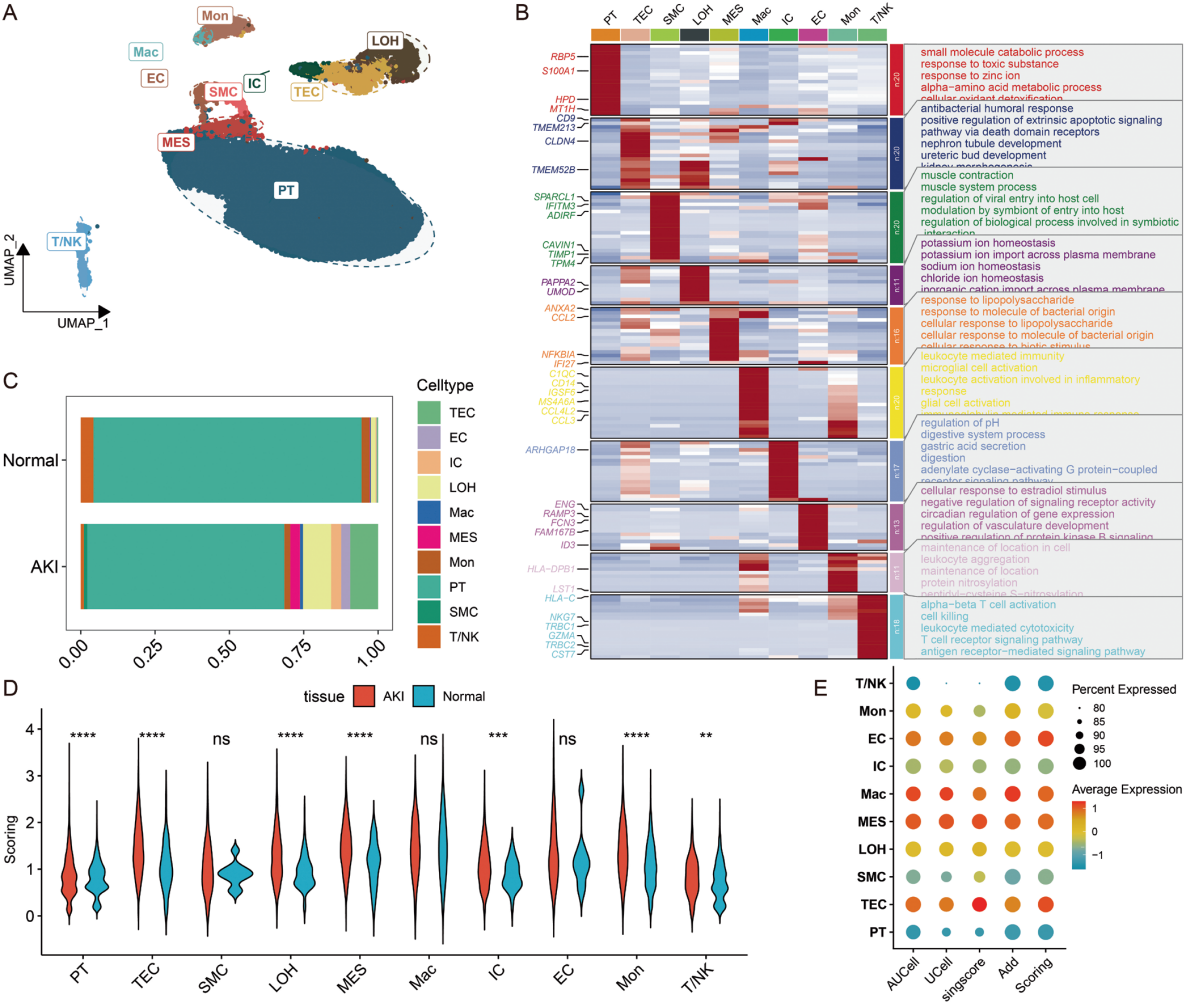
AKI single‐cell landscapes. (A) Single‐cell UMAP plot. (B) Differences in cell proportions between normal and AKI groups. (C) Heatmap of genetic and functional enrichment of each cell type marker. (D) Differences in mtPCD scores in AKI and normal tissues. (E) Visualisation of mtPCD scores in different cell types.

We identified ten distinct cell types using cell annotation based on marker genes and differentially expressed genes across clusters. These include Tubular Epithelial Cells (TEC), Interstitial Cells (IC), Macrophages (Mac), Monocytes (Mon), Natural Killer T (T/NK) Cells, Smooth Muscle Cells (SMC), Loop of Henle Cells (LOH), Proximal Tubule Cells (PT), Endothelial Cells (EC) and Mesangial Cells (MES) (Figure 1A). We then performed GO/KEGG analysis to determine enriched pathways associated with each cell type and visualised the marker genes for each cell type. Additionally, the outcomes aligned with each cell type's biological functions (Figure 1B). We subsequently compared the differences in cell proportions between the normal group and the AKI group. The results exhibited a significant increase in the proportion of cells, including TEC, in the AKI group compared to the normal group (Figure 1C).

Next, we utilised four scoring methods: AUCell, UCell, singscore and Addmodelscore to score 24 key mtPCD genes. These four scores were combined and standardised to integrate the scores obtained from various algorithms, resulting in the scoring. We compared the expression of the mtPCD score across different groups. The analysis indicated that the mtPCD score was significantly higher in the AKI group than in the normal group in various cell types, including PT, TEC, LOH, MES, IC, Mon and T/NK (Figure 1D). Subsequently, we visualised the scoring for each cell type. TEC cells exhibited significant differences and had the highest scores (Figure 1E), indicating that TEC may be a key cell population in AKI where mitochondrial and cell death pathways intersect.

### 3.2 Identification and Functional Analysis of mtPCD TEC Subgroups

**FIGURE 2:**
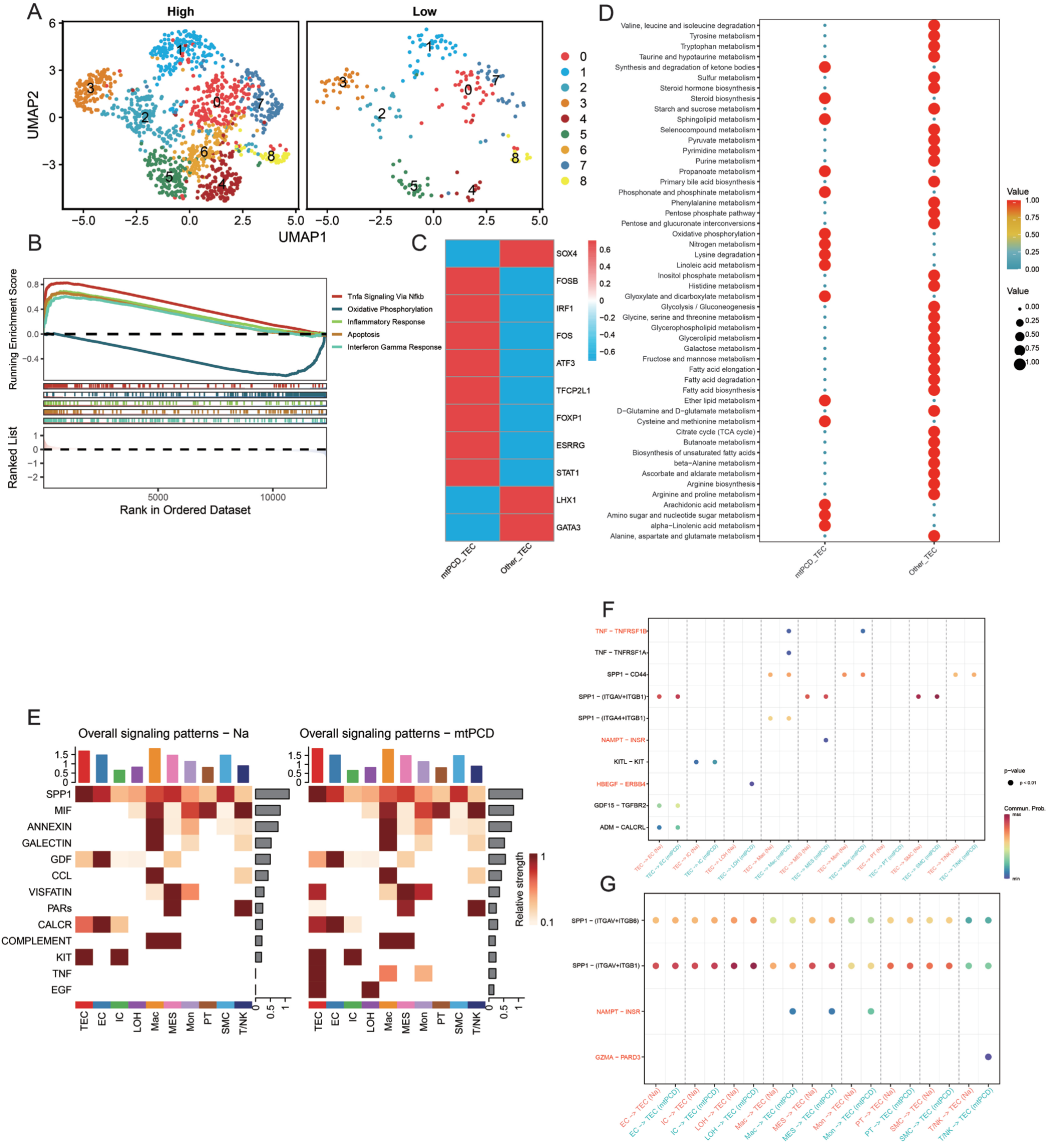
Identification and functional analysis of mtPCD‐related TEC subpopulations. (A) Visualisation of UMAP in high and low mtPCD groups. (B) Analysis of GSEA differences in high and low mtPCD groups. (C) Analysis of SCENIC in high and low mtPCD groups. (D) Analysis of metabolic differences in high and low mtPCD groups. (E) Heatmap of cellular communication differences. (F) MtPCD TEC as ligand cell communication bubble graph. (G) MtPCD TEC as receptor cell communication bubble graph.

To determine the role of mtPCD in TEC cells, we divided the cells into high and low‐expression groups based on the median of mtPCD scoring. We extracted TEC subgroups for further dimensional reduction and clustering. The results indicated that the cluster 6 subgroup was exclusively in the high‐mtPCD scoring group. This suggests that the cluster 6 subgroup may be the key subgroup directly involved in mtPCD. Therefore, we defined cluster 6 as mtPCD‐TEC and the other subgroups as Other‐TEC (Figure 2A). A single‐cell GSEA was performed to determine key pathway differences between mtPCD‐TEC and Other‐TEC. We visualised the top five pathways and found that compared to Other‐TEC, mtPCD‐TEC exhibited higher expression in pathways, including TNF, interferon gamma response, apoptosis and immune response, all of which are directly associated with mtPCD (Figure 2B). Furthermore, SCENIC analysis revealed transcriptional differences between the two, with mtPCD‐TEC significantly overexpressing transcription factors directly linked to cell death and inflammatory pathways, including FOSB, IRF1, FOS, ATF3, TFCP2L1, FOXP1, ESRRG and STAT1 (Figure 2C). Metabolic analysis indicated that mtPCD‐TEC has a higher level of oxidative phosphorylation and different glycolytic levels than Other‐TEC (Figure 2D).

Using cell communication analysis, we investigated the differences between mtPCD‐TEC and Other‐TEC in detail. The results revealed that mtPCD‐TEC has unique pathways in communication with other cell subgroups, including TNF, EGF, PARs, VISFATIN and a higher intensity of CALCR (Figure 2E). When TEC cells serve as ligands, there exists an HBEFG‐ERBB4 ligand‐receptor pair between mtPCD‐TEC and LOH, a NAMPT‐INSR ligand‐receptor pair between mtPCD‐TEC and MES and TNF‐TNFRSF1B ligand‐receptor pairs between mtPCD‐TEC and both Mac and Mon. This indicates that the TNF signalling pathway can influence cell communication between mtPCD‐TEC and myeloid cells (Figure 2F). When mtPCD‐TEC acts as the receptor, Mac, Mon, and PT cells all affect mtPCD‐TEC through NAMPT‐INSR, and T/NK cells affect mtPCD‐TEC through GZMA‐PARD3 (Figure 2G). This suggests that mtPCD‐TEC has a unique mode of cell communication and distinct ligand–receptor interactions.

### 3.3 hdWGCNA Analysis of TEC

**FIGURE 3:**
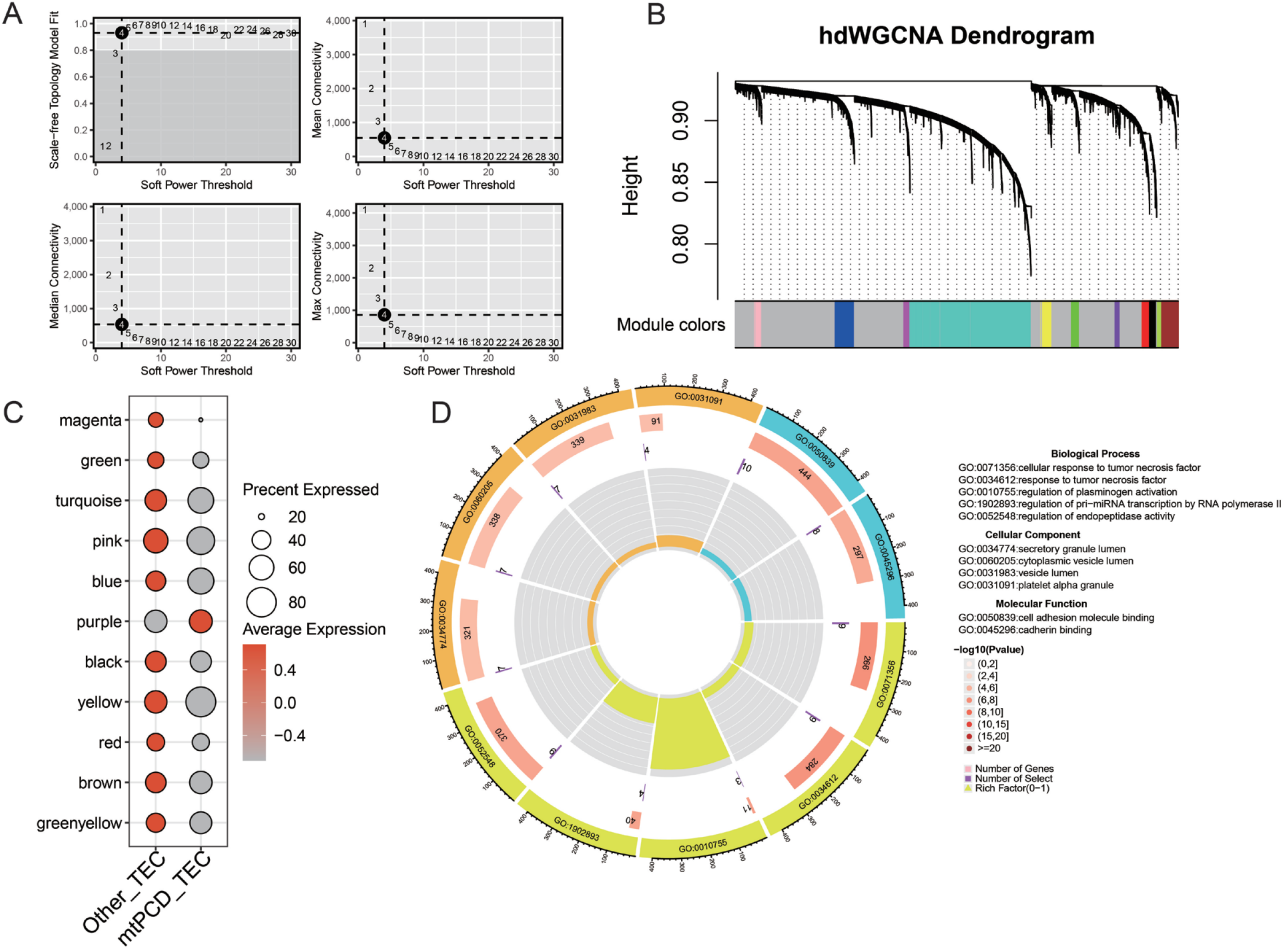
hdWGCNA analysis. (A) Soft threshold. (B) Clustering diagram. (C) Degree of enrichment of each module in the cluster. (D) Analysis of functional enrichment of genes in the purple module.

Based on the differences in TEC, we extracted AKI samples and performed hdWGCNA analysis on the TEC subgroups within AKI to determine the association with AKI. We set the soft threshold to 4 (Figure 3A) and conducted clustering for the DT subgroup (Figure 3B). This resulted in the identification of genes across 11 modules. We then visualised the enrichment of each module in the cluster. The results revealed that the purple module was enriched only in MtPCD‐TEC (Figure 3C). We extracted the purple module, comprising 87 genes. The subsequent functional enrichment analysis indicated that its functions primarily involve cellular response to tumour necrosis factor, secretory granule lumen, and cell adhesion molecule binding (Figure 3D).

### 3.4 Development of a Diagnostic Model

**FIGURE 4:**
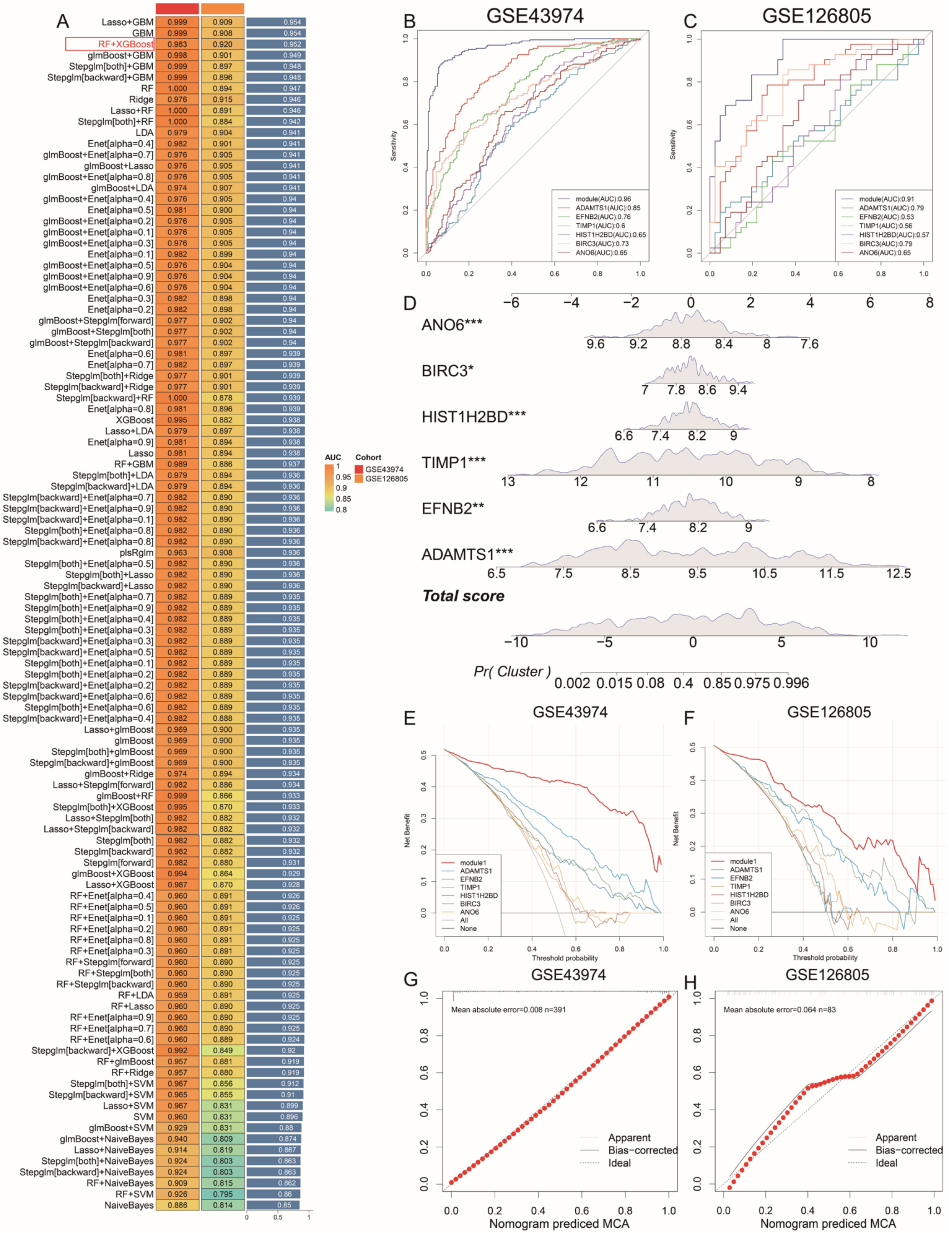
Machine learning for construction and evaluation of diagnostic models. (A) Diagnostic model construction. (B) GSE43974 AUC curve. (C) GSE126805 AUC curve. (D) Nomogram plot. (E) GSE43974 decision curve. (F) GSE126805 decision curve. Calibration plots for the nomogram in GSE43974 (G; *n* = 391) and GSE126805 (H; *n* = 83).

We intersected the 87 genes from the purple module with differentially expressed genes in the GSE43974 dataset to identify key module genes. Subsequently, we integrated 10 machine‐learning algorithms and 108 algorithm combinations for further analysis. We utilised GSE43974 as our training dataset and GSE126805 for validation purposes. The models were evaluated and selected based on their area under the curve (AUC) and concordance index (c‐index) performance. In balancing the number of genes in each model with their validation effectiveness, the top three models emerged as Lasso+GBM, GBM, and RF + XGBoost. Considering the insignificant differences in their c‐index scores and prioritising gene count efficiency, we identified RF + XGBoost as the superior diagnostic model (Figure 4A). This model includes a powerful set of six diagnostic genes: ADAMTS1, EFNB2, TIMP1, HIST1H2BD, BIRC3 and ANO6. Notably, this model exhibited superior performance in the validation set compared to other models, reinforcing its status as the most robust and reliable diagnostic tool. Further validation was performed through receiver operating characteristic (ROC) curve analysis across both datasets, confirming the diagnostic model's exceptional accuracy and superiority over individual gene assessments (Figure 4B,C).

Furthermore, we developed a nomogram based on the diagnostic model, which exhibited significant diagnostic accuracy (Figure 4D). The decision curve analysis of the model in both the training and validation sets indicated superior performance compared to single genes (Figure 4E,F). In GSE43974 (*n* = 391), the bias‐corrected calibration curve almost perfectly overlaps the 45° ideal line across all deciles, yielding a mean absolute error (MAE) of 0.008 (Figure 4G). In the smaller GSE126805 cohort (*n* = 83), calibration remains acceptable (MAE = 0.064), with only modest deviation from the ideal line around predicted probabilities of 0.4–0.7 (Figure 4H).

### 3.5 Expression Patterns of Key Genes and Their Correlation With Immune Infiltration and Signalling Pathways in AKI

**FIGURE 5:**
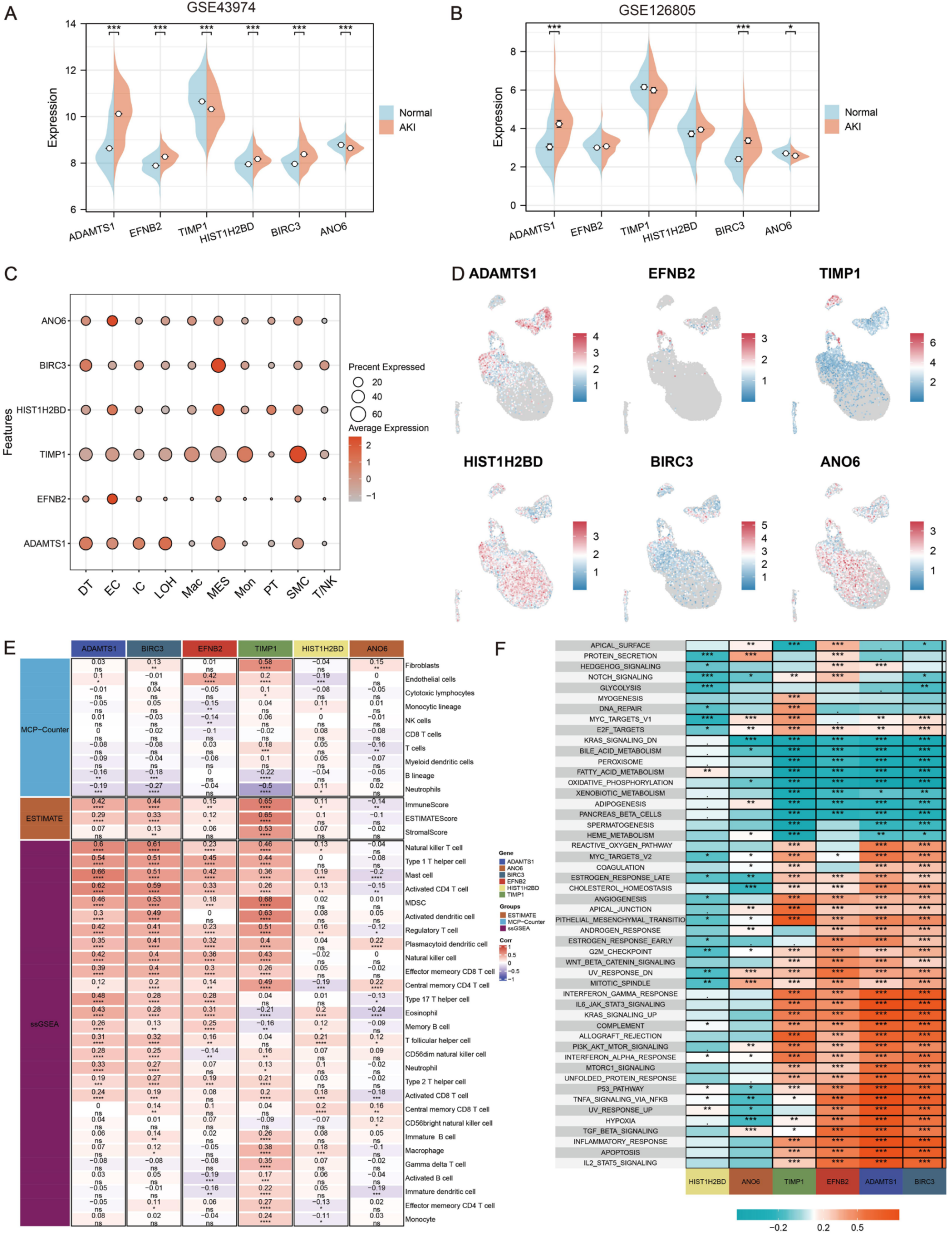
The localization of key genes and the relationship of key genes with immunity and pathways. (A) Difference in GSE43974 expression. (B) Difference in GSE126805 expression. (C) Key genes expression in single cells feature plot. (D) Key genes expression in single cells UMAP plot. (E) Correlation of key genes with immunity scores in the training set. (F) Correlation of key genes with pathways in the training set (*p < 0.05, **p < 0.01, ***p < 0.001, ****p < 0.0001).

To further identify the expression patterns of genes in our model, we determined the differences in expression of six model genes within the GSE43974 and GSE126805 datasets. The analysis revealed that only ADAMTS1, BIRC3 and ANO6 had expression differences in both datasets. Remarkably, BIRC3 and ADAMTS1 were significantly upregulated in the AKI groups of the two datasets, whereas ANO6 exhibited a significant decrease in expression in the AKI groups (Figure 5A,B). We utilised dot plot and feature plot visualisations exhibiting a significant enrichment of ADAMTS1 in TEC (Figure 5C,D).

Furthermore, we employed methods, including ESTIMATE, MCP‐counter and ssGSEA, to determine the level of immune infiltration in AKI samples. Subsequently, we calculated and visualised the Pearson correlation between our six key genes and these immune scores. Combining the earlier differential analysis results, significant positive correlations were observed between ADAMTS1, BIRC3 and a series of immune cells, including natural killer T (T/NK) cells (Figure 5E). Moreover, we analysed the correlations between the model genes and key pathways. The results revealed that the key genes had a significantly positive correlation with pathways, including the TNF signalling pathway, apoptosis, hypoxia, TGF‐β signalling pathway and the JAK‐STAT3 signalling pathway (Figure 5F).

### 3.6 Identification of ADAMTS1 as a Key Gene in AKI

**FIGURE 6:**
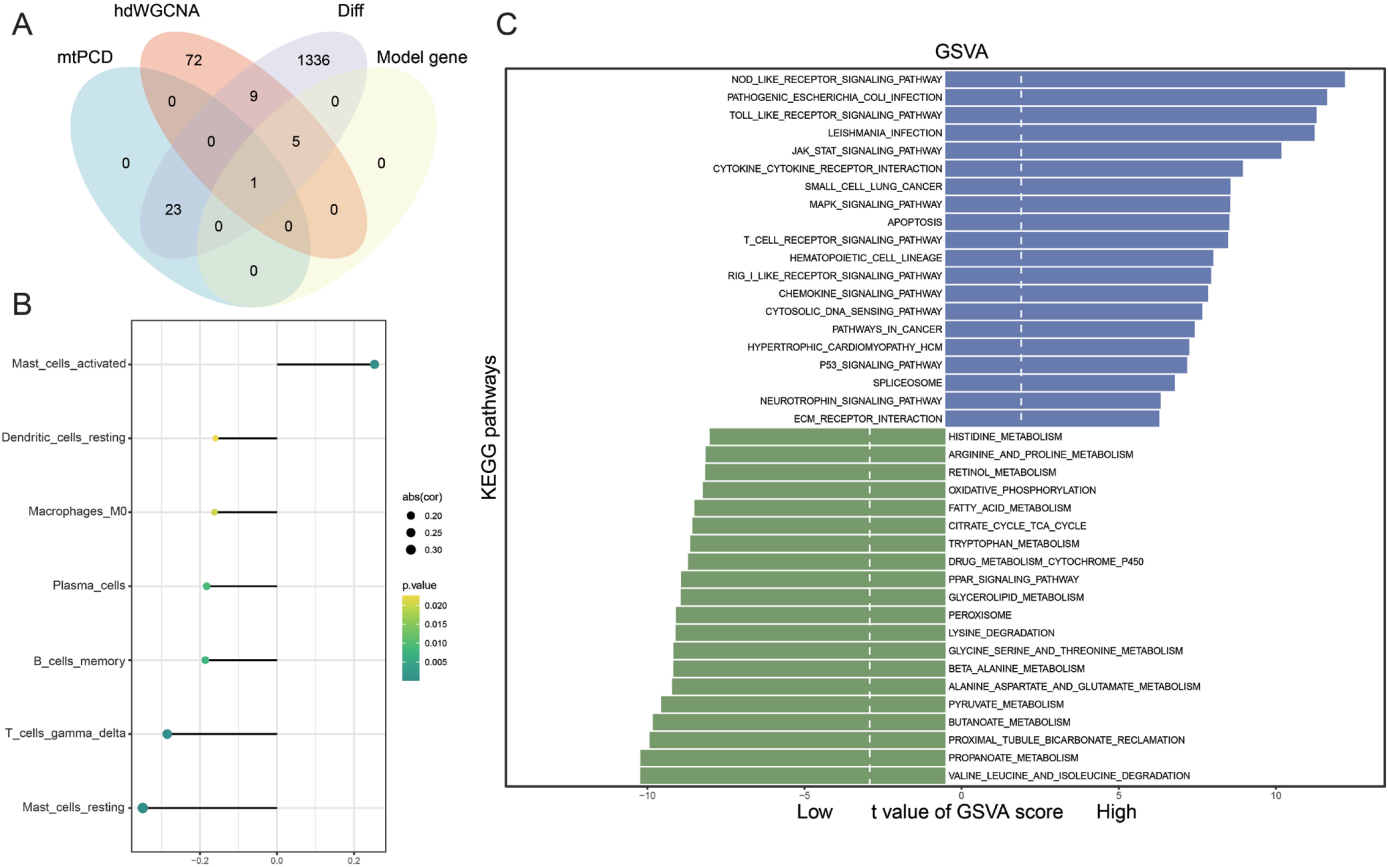
Single‐gene analysis of key model genes. (A) Key gene screening Wayne plots. (B) Single‐gene immune infiltration analysis. (C) Single‐gene GSEA analysis.

We intersected mtPCD interplay genes, hdWGCNA purple module genes, differential genes, and model genes, ultimately identifying ADAMTS1 as the most critical gene (Figure 6A). Subsequently, we performed a CIBERSORT analysis on the high‐ and low‐expression groups of ADAMTS1 based on GSE43974. The results suggested a negative correlation of ADAMTS1 with most functional immune cells, indicating its potential role in immune suppression (Figure 6B). Moreover, GSVA analysis revealed that the high‐expression group of ADAMTS1 was significantly enriched in pathways, including TNF, interferon‐gamma response and apoptosis (Figure 6C).

### 3.7 Drug Prediction and Molecular Docking

**FIGURE 7:**
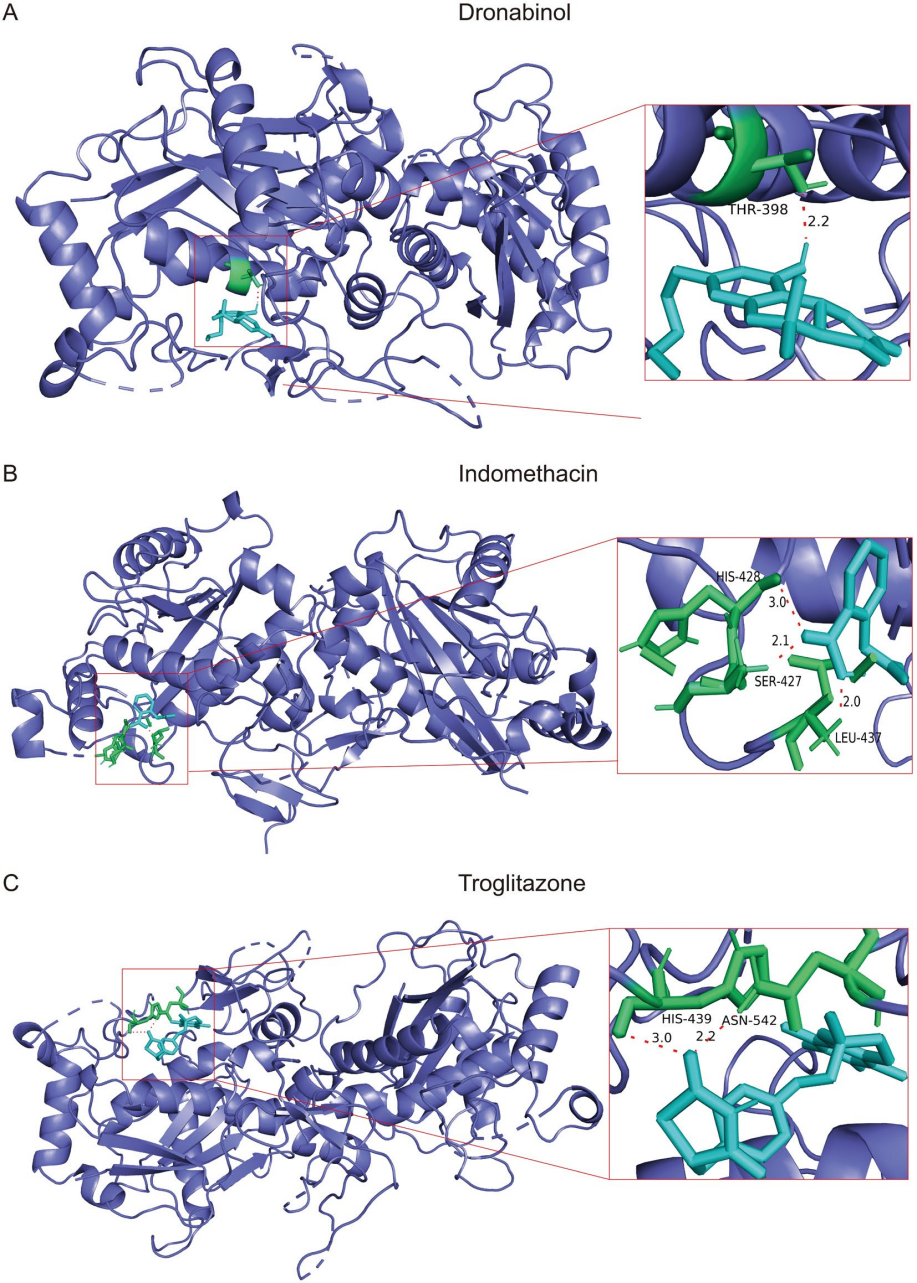
Drug prediction and molecular docking. (A) Dronabinol. (B) Indomethacin. (C) Troglitazone.

For the key gene, ADAMTS1, in our prognostic model, we screened for drugs that can affect the expression of ADAMTS1 using CTD. We obtained the structures of key drugs from PubChem and selected three drugs that can reduce gene expression (dronabinol, indomethacin and troglitazone). We performed molecular docking for these drugs, and the binding energies found were 6.59 kJ/mol for dronabinol, 4.11 kJ/mol for indomethacin and 7.05 kJ/mol for troglitazone. Finally, we used Pymol to visualise molecular docking (Figure 7A–C).

### 3.8 Reduction of ADAMTS1 Expression Alleviates Renal Injury in I/R Mice by Suppressing Inflammatory Responses

**FIGURE 8:**
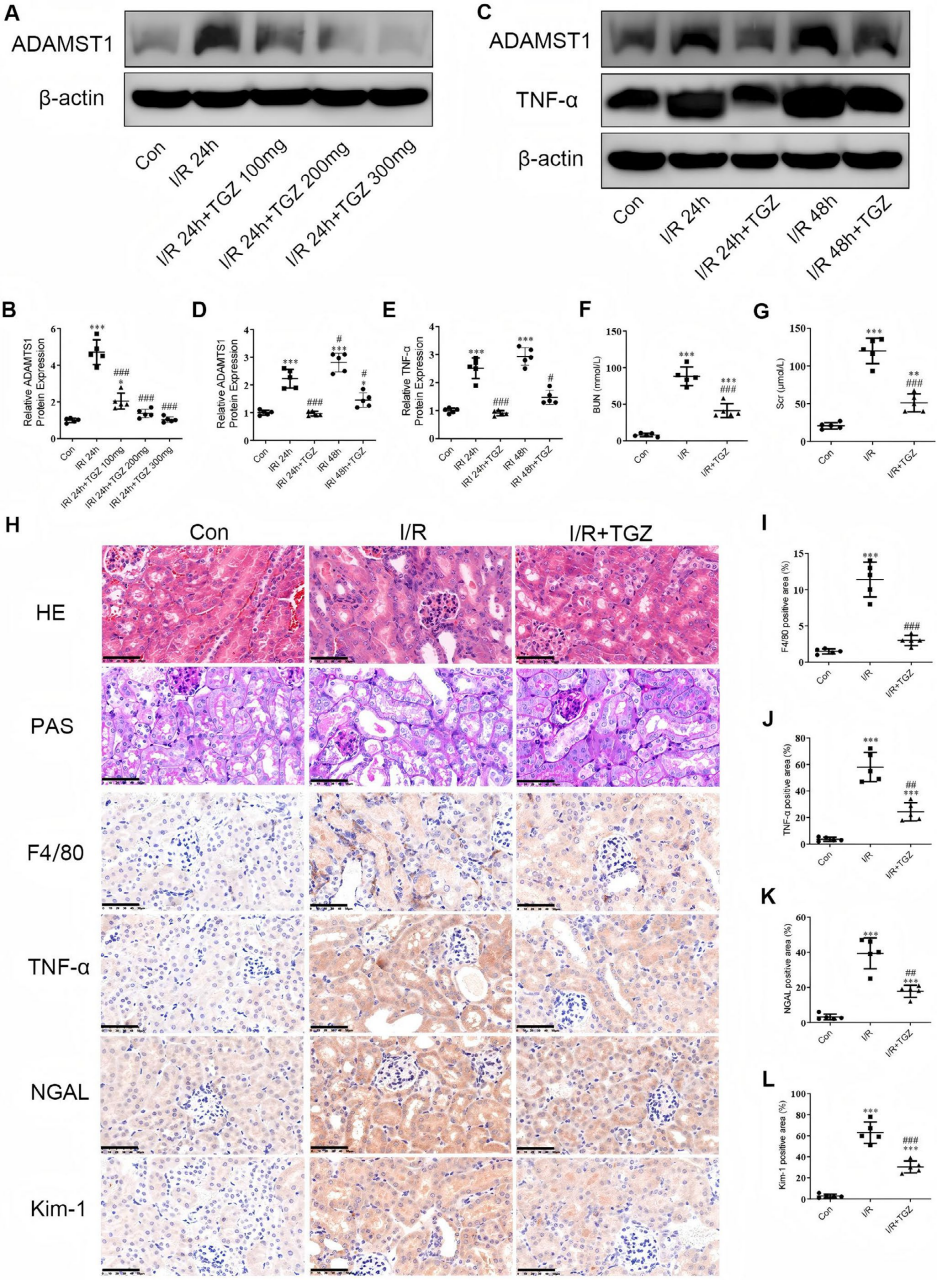
Reduction of ADAMTS1 expression alleviates renal injury in I/R mice. (A–E) Detection of ADAMTS1 and TNF‐α levels using Western blotting and selection of the optimal concentration of TGZ and the optimal time point for ischemia–reperfusion injury. (F and G) Evaluation of Scr and BUN levels in all mice. (H–L) Representative images of mouse kidney histology using HE and PAS staining; evaluation of TNF‐α, F4/80, NGAL, and Kim‐1 protein levels in mouse kidneys using IHC staining. Scale Bar = 50 μm. *p‐value < 0.05; **p‐value < 0.01; ***p‐value < 0.001 to the control group; ^#^ *p* < 0.05, ^##^ *p* < 0.01, ^###^ *p* < 0.001 to the I/R group.

We first determined the optimal dose of Troglitazone (TGZ) in mice. The results exhibited a significant increase in ADAMTS1 protein expression in I/R mice (Figure 8A,B). Treatment of TGZ at 100 mg reduced ADAMTS1 levels compared to that in the I/R group, with a significant reduction observed in the TGZ 200 mg group. However, the difference in ADAMTS1 expression between the TGZ 200 mg and TGZ 300 mg groups remained insignificant. Consequently, we selected an oral dose of 200 mg TGZ for five weeks for the subsequent experiments. The expression of ADAMTS1 and TNF‐α were increased in the I/R 24 h group compared to the control group, with a significant increase in the I/R 48 h group (Figure 8C–E). Administration of TGZ significantly inhibited the expression of ADAMTS1 and TNF‐α. In the subsequent experiments, we used I/R 48 h to determine pathological changes in mouse kidneys. BUN and SCr levels were elevated in the I/R group compared to the control group, with significant improvement in the TGZ‐treated mice (Figure 8F,G). I/R mice exhibited severe renal tubulointerstitial injury, including tubular dilation, atrophy and inflammatory cell aggregation (Figure 8H). TGZ treatment significantly alleviated these injuries. Immunohistochemistry results exhibited positive staining for the macrophage marker F4/80 and the inflammatory factor TNF‐α in I/R kidneys, indicating interstitial infiltration of inflammatory cells (Figure 8I,J). Furthermore, the expression levels of tubular injury markers, including Kim‐1 and NGAL, were elevated in I/R kidneys compared to the control group. TGZ treatment significantly reduced the expression of Kim‐1 and NGAL proteins (Figure 8K,L). Our results suggest that TGZ alleviates renal injury in I/R mice by reducing ADAMTS1 expression and suppressing renal inflammatory responses.

### 3.9 Reduction of ADAMTS1 Expression Inhibits Mitochondria‐Mediated Renal Apoptosis in I/R Mouse Model

**FIGURE 9:**
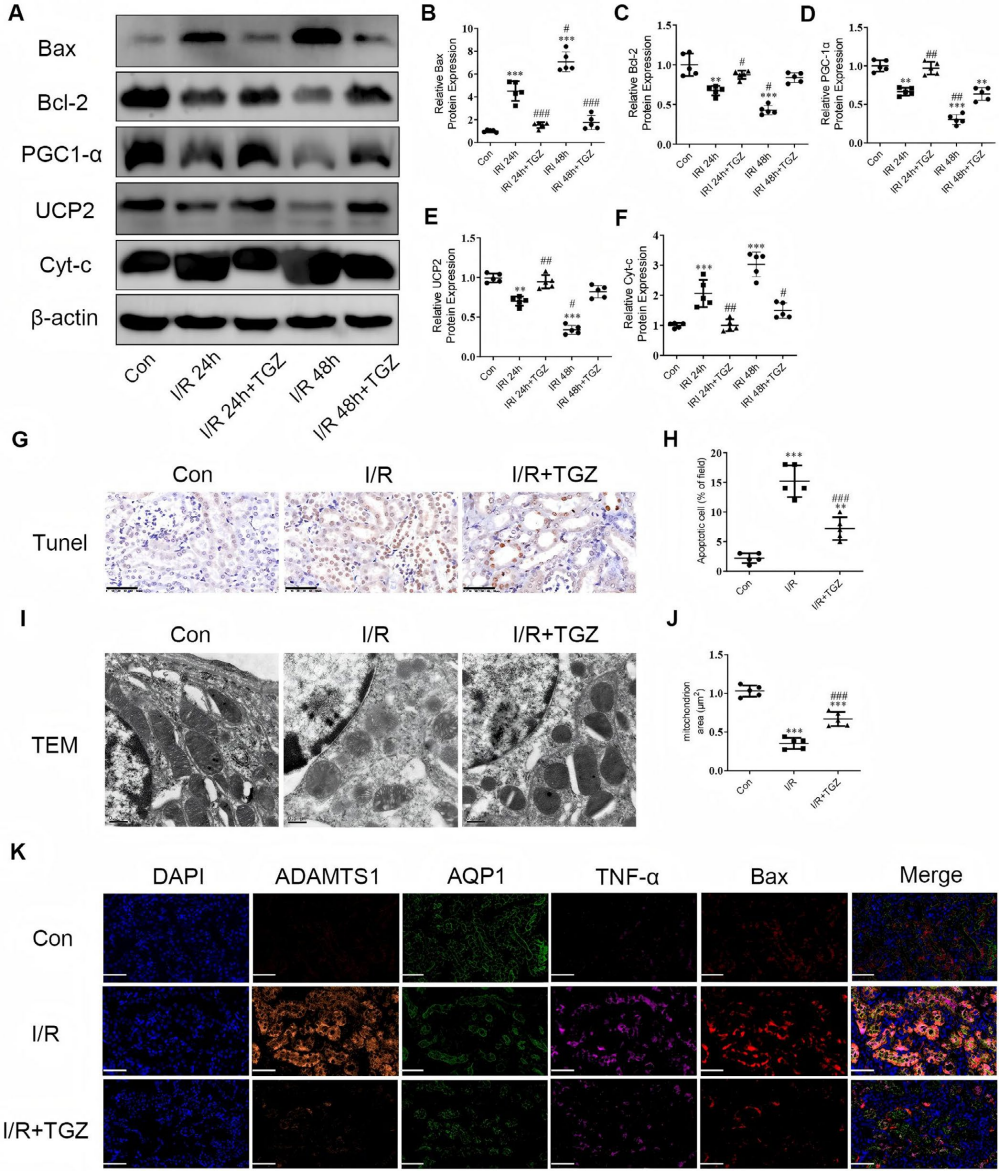
Reduction of ADAMTS1 expression inhibits mitochondria‐mediated renal apoptosis in I/R mice. (A–F) Detection of Bax, Bcl‐2, PGC1‐α, UCP2, and Cyt‐c levels using Western blotting. (G and H) TUNEL staining of mouse kidney tissues, scale bar = 50 μm. (I and J) Electron microscopy of mouse kidney tissues, scale bar = 0.5 μm. (K) Immunofluorescence detection of ADAMTS1, AQP1, TNF‐α, and Bax protein levels in mouse kidney tissues. Scale bar = 50 μm. **p‐value < 0.01; ***p‐value < 0.001 to the control group; ^#^ *p* < 0.05, ^##^ *p* < 0.01, ^###^ *p* < 0.001 to the I/R group.

The expression of mitochondrial biogenesis proteins PGC1‐α and UCP2 and apoptosis proteins Bax, Bcl‐2, and Cyt‐c in mouse kidneys was first determined. Bax and Cyt‐c protein levels increased in the I/R 24 h group, whereas Bcl‐2, PGC1‐α, and UCP2 levels decreased. These trends were more significant in the I/R 48 h group. Administration of TGZ significantly reversed the expression of these proteins (Figure 9A–F). Consequently, we continued to use the I/R 48 h model in subsequent experiments. We utilised the terminal deoxynucleotidyl transferase dUTP nick‐end labeling (TUNEL) assay to detect apoptotic cells in kidney tissues. An increase in apoptotic cells post‐I/R surgery was observed, primarily in the tubular area (Figure 9G,H). The I/*R* + TGZ group's samples exhibited fewer TUNEL‐positive cells than those in the I/R group. Furthermore, TEM images indicated that the mitochondria in the I/R group were smaller, with increased mitochondrial membrane density and loss of cristae. These changes were reversed after TGZ treatment (Figure 9I,J). The immunofluorescence results revealed that the localization of ADAMTS1, TNF‐α, F4/80, and Bax proteins was significantly enhanced in the kidneys of I/R mice, while the localization of the tubular marker protein AQP1 was reduced. TGZ treatment effectively reversed these changes (Figure 9K). Our study confirms that TGZ treatment can significantly inhibit ADAMTS1 expression and mitigate renal injury by suppressing mitochondria‐mediated tubular apoptosis.

### 3.10 ADAMTS1 Expression Is Significantly Increased in AKI, Accompanied by Activated Mitochondria‐Mediated Apoptosis

**FIGURE 10:**
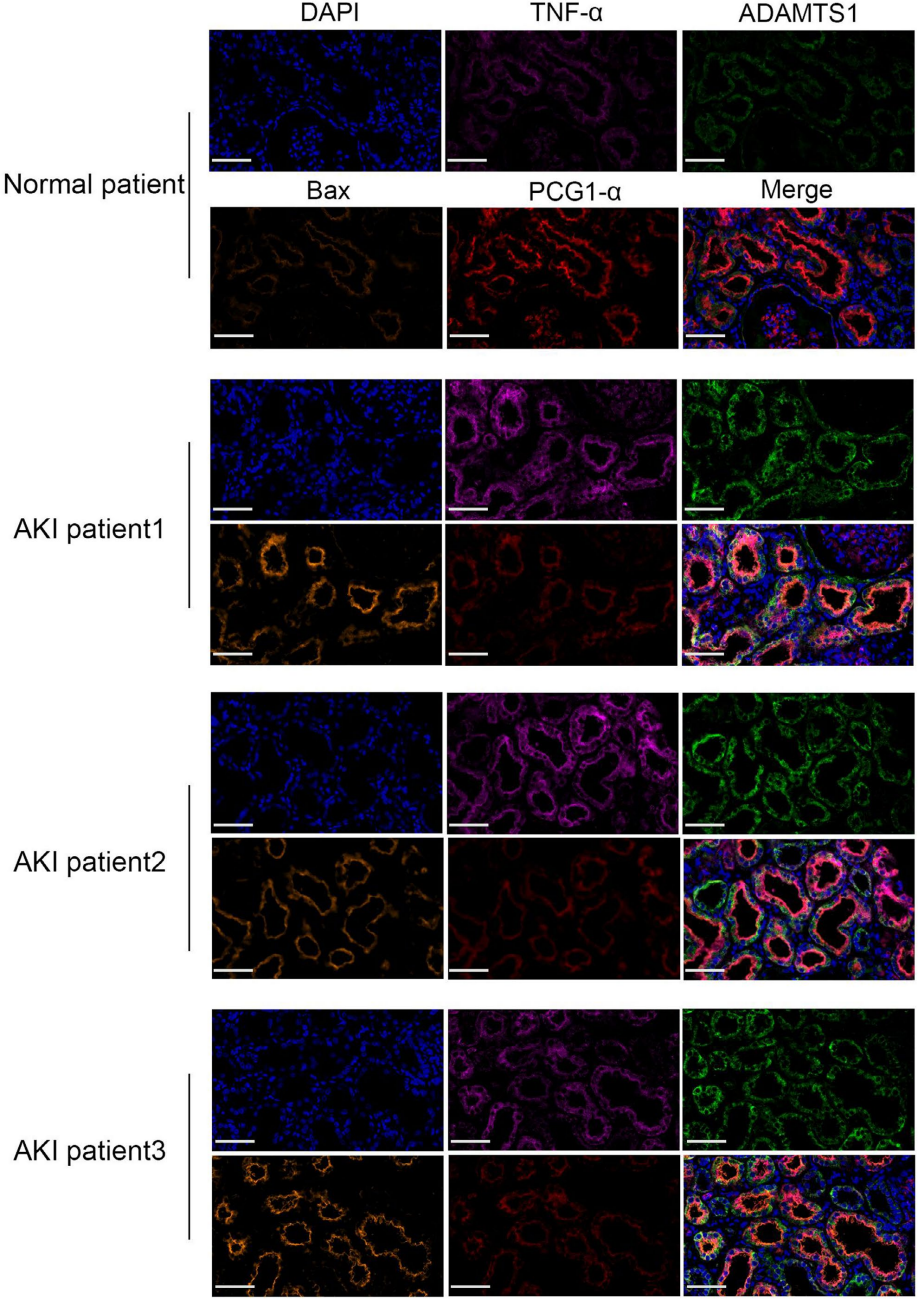
Immunofluorescence detection of ADAMTS1, TNF‐α, PGC1‐α, and Bax protein levels in kidney tissues of AKI patients. Scale Bar = 50 μm.

We observed ADAMTS1 expression in kidney samples from AKI patients using immunofluorescence. Immunofluorescence revealed significantly higher ADAMTS1 protein levels in the kidney tissues of AKI patients than in the control group, with significant enrichment in the tubular areas. TNF‐α and Bax protein levels were elevated, whereas PGC1‐α protein levels decreased in AKI kidney tissues (Figure 10). These results validate ADAMTS1's role in AKI pathogenesis.

## 4 Discussion

In this study, we used high‐resolution single‐cell sequencing technology to extensively investigate the renal microenvironment in AKI, with a particular focus on TEC, a cell type that plays a crucial role in the progression of AKI. Moreover, we identified a TEC subtype, mtPCD TEC, with key biological functions. The unique role of this subtype in AKI was revealed by its significant differences from other TEC subtypes in regulating metabolic pathways and transcription factor activity. The characteristics of the mtPCD TEC subtype were analysed to develop a diagnostic model that included six key genes with high predictive value and clinical relevance. This model improves the accuracy of AKI diagnosis and provides new tools for clinical decision‐making. Finally, through further screening and validation, we identified ADAMTS1 as a novel therapeutic target for AKI and determined its effective targeted intervention drugs. These drugs were preliminarily validated in vivo animal models. Overall, this study has developed a new diagnostic model for AKI and identified ADAMTS1 as a novel therapeutic target for AKI, providing new insights into the AKI diagnosis and treatment.

Recently, research on machine learning predictive models for AKI has been extensive, focusing on data from three public databases: MIMIC‐III, AmsterdamUMCdb and eICU. These databases primarily include patient vital signs at admission, laboratory tests and other clinical indicators measured after admission. However, the results indicated that 57% of patients meet the AKI criteria on the first day of ICU admission, which results in poor predictive capabilities for early‐onset AKI. Moreover, although most models can moderately differentiate AKI events with AUC values ranging from 0.7 to 0.9, they have not undergone effective external validation, which limits their generalisability and applicability to larger study populations. Multiple machine learning approaches suggest that selecting better models to improve diagnostic precision is essential, as some studies have integrated methods such as logistic regression. In our study, we developed a new AKI diagnostic model based on the gene expression profiles of AKI patients, integrating several advanced machine learning methods. This model demonstrated good diagnostic accuracy in both training and validation sets. We hypothesised that gene expression profiles can indicate the onset of disease before clinical symptoms appear, thus aiding in earlier diagnosis and intervention. Furthermore, models based on gene expression can help identify patients who can respond to specific treatments or experience side effects. Additional gene expression data analysis identified new disease‐related genes or combinations of genes, which can be potential diagnostic or therapeutic targets in the future.

Inflammatory cascades play a crucial role in the pathophysiology of I/R renal injury, causing tissue damage through various mediators [12]. I/R activates immune cells and increases inflammatory factors such as TNF‐α, which infiltrate the interstitium by enhancing cell adhesion. Moreover, tubular epithelial cells produce pro‐inflammatory cytokines (TNF‐α and IL‐1β) and chemokines (sMCP‐1), promoting the inflammatory response [13]. In vivo models revealed that I/R significantly increased serum Scr and BUN levels in mice, which are markers of AKI. Troglitazone treatment reduced these levels, inhibiting cell infiltration and renal damage. Our experiments indicated that troglitazone reduced ADAMTS1 and pro‐inflammatory cytokines in I/R mice while increasing tubular markers NGAL and Kim‐1. This suggests that troglitazone may improve I/R renal injury by reducing ADAMTS1 protein levels, inhibiting tubular inflammatory responses. Being a key immune cell, macrophages are closely involved in AKI development and progression [14]. Tubular interstitial macrophage accumulation is associated with tissue repair and immune responses. F4/80, a mature cell surface glycoprotein, is a specific biomarker for mouse macrophages [15]. In this study, we measured F4/80 protein levels and found that troglitazone treatment reduced F4/80 infiltration in I/R mice compared to I/R‐AKI mice, indicating troglitazone inhibits macrophage accumulation in the I/R‐AKI model.

Renal tubular epithelial cells have high energy demands and are metabolically active, making them primary targets for injury [16]. Studies suggested that mitochondrial dysfunction and apoptosis in renal tubular epithelial cells are crucial in AKI development [17]. Apoptosis is a programmed cell death process mediated by various transcripts, frequently occurring as the mitochondrial pathway's downstream regulations, representing a classic apoptosis signalling route. Dysregulated heterodimer Bax/Bcl‐2 in mitochondria results in the upregulated Cyt‐c, which activates downstream apoptosis‐related caspases, further promoting the apoptosis process [18]. Our experiments indicate that troglitazone administration in mice reduced ADAMTS1 expression levels in I/R‐AKI kidneys. Consequently, the levels of mitochondrial pathway apoptosis proteins Bax and Cyt‐c were reduced, while the levels of mitochondrial biogenesis proteins PGC1‐α and UCP2 were increased. Our study observed reduced mitochondrial cristae, increased membrane density and decreased volume in the kidney tissue from I/R‐AKI mice. Troglitazone alleviated mitochondrial damage in I/R‐AKI mice by inhibiting ADAMTS1 expression. These findings suggest that troglitazone can mediate mitochondrial pathway‐induced tubular cell apoptosis by inhibiting ADAMTS1, thus reducing inflammation and kidney damage in I/R‐AKI model mice.

The ADAMTS family of metalloproteinases is associated with various physiological and pathological conditions. Among the 19 proteases in this family, ADAMTS1, as the first member, has received considerable attention for its role in multiple diseases [19]. Elevated levels of ADAMTS1 can promote tumorigenic changes, including increased tumour cell proliferation [20], inhibition of apoptosis and altered angiogenesis [21]. In addition, it significantly facilitated the remodelling of the extracellular matrix environment, thereby facilitating tumour progression and metastasis [19, 22]. However, some studies have reported ADAMTS1 as a tumour suppressor [23]. Systemic lipopolysaccharide‐induced inflammation has resulted in a significant increase in ADAMTS1 levels. Besides, ADAMTS1 was identified as an early hypoxia‐responsive gene; however, the significance of hypoxia‐induced increased expression of ADAMTS1 has not been fully demonstrated [24]. The role of ADAMTS1 in AKI has been suggested as an inhibitor of VEGF, which can result in inadequate vascular repair responses following ischemia–reperfusion injury. This could make the kidneys more susceptible to chronic disease. These findings indicate that different conditions can induce ADAMTS1 expression. Furthermore, it has been reported that in the human chondrosarcoma cell lines C3842 and OUMS‐27, the inflammatory stimulus IL‐1β reduced ADAMTS1 mRNA levels, while hypoxia did not alter ADAMTS1 mRNA in C3842 [25]. This suggests that ADAMTS1 expression depends on the stimulus and cell type; however, this requires further investigation. In our study, considering that both hypoxia and inflammation are key factors in the AKI process and are interrelated, combined with our experimental results, we propose that the unique hypoxic and inflammatory microenvironment formed in the renal microenvironment during AKI is the primary reason for the ADAMTS1 upregulation in tubular epithelial cells. Furthermore, ADAMTS1 accelerates the progression of AKI by promoting mitochondrial pathway apoptosis in tubular epithelial cells. Troglitazone can significantly alleviate the progression of AKI by inhibiting ADAMTS1‐mediated tubular epithelial cell injury. Therefore, targeting ADAMTS1 as an early diagnostic and potential therapeutic target for AKI is important. However, troglitazone has potential hepatotoxic side effects [26]. Further in‐depth studies on its mechanism of action on ADAMTS1 to alleviate AKI or using combination therapy to reduce its adverse effects could also provide valuable insights for the alleviation and treatment of AKI.

Although our study highlights key cellular subgroups in the process of AKI and proposes new therapeutic targets, there are a few limitations. The identified key cellular subgroup requires further analysis and biological validation to accurately describe their biological functions and roles in AKI. Moreover, validation in large‐scale population studies is required before the diagnostic model can be implemented in future practical settings. In summary, we emphasise the importance of tubular epithelial cells in the AKI process. Our study endorses the continued development of protective agents for tubular epithelial cells, as the stress‐induced injury and death of tubular epithelial cells are critical events in the onset and progression of kidney diseases. Additionally, the therapeutic effects of various agents that inhibit tubular epithelial cell death have been demonstrated in AKI. Our study identified a set of TEC subgroups associated with AKI progression and proposed an effective diagnostic model, providing new insights for AKI diagnosis and treatment. Finally, our findings highlight ADAMTS1 as a promising biomarker and effective molecular target for AKI treatment. Future drug improvements based on ADAMTS1 and the development of new small‐molecule drugs are highly attractive and could provide a promising therapeutic approach for AKI treatment.

## Author Contributions

**Qiming Gong:** conceptualization (equal), data curation (equal), formal analysis (equal), funding acquisition (equal), writing – original draft (equal), writing – review and editing (equal). **Yakun Wang:** funding acquisition (equal), validation (equal), writing – original draft (equal), writing – review and editing (equal). **Fahui Liu:** resources (equal), software (equal), validation (equal), writing – original draft (equal), writing – review and editing (equal). **Tingting Zhou:** visualization (equal), writing – original draft (equal), writing – review and editing (equal). **Mengqin Tu:** formal analysis (equal), investigation (equal), methodology (equal). **Junle Li:** data curation (equal), investigation (equal), methodology (equal). **Wei Huang:** visualization (equal), writing – original draft (equal), writing – review and editing (equal). **Xu Lin:** writing – original draft (equal), writing – review and editing (equal). **Wenjuan Sun:** visualization (equal), writing – original draft (equal), writing – review and editing (equal).

## Ethics Statement

All procedures conducted in studies involving human participants adhered strictly to the ethical standards set forth by the institutional and national research committees, as well as the principles outlined in the 1964 Helsinki Declaration and its subsequent amendments or comparable ethical standards. This ensures that the rights and welfare of participants are protected throughout the research process. The study received formal approval from Shanghai Sixth People's Hospital (Clinical Trial Number: V1.0/No. 20240601). In addition, all animal procedures carried out during the study were thoroughly reviewed and approved by the clinical experiment ethics committee of the Animal Experiment Ethics Committee of Youjiang Medical University for Nationalities (Ethical batch number: 2021103001). It is imperative that such approvals are obtained to ensure that the experiments are conducted ethically and responsibly. Furthermore, all animal procedures complied with the National Institutes of Health Guidelines for the Care and Use of Laboratory Animals, as well as the ARRIVE Guidelines, which provide a framework to promote transparency and reproducibility in animal research. This commitment to ethical standards reflects a strong dedication to both human and animal welfare within the context of scientific research.

## Consent

The authors have nothing to report.

## Conflicts of Interest

The authors declare no conflicts of interest.

## Supporting information


**Appendix S1:** jcmm70801‐sup‐0001‐Supinfo.pdf.

## Data Availability

The data are available from the authors upon reasonable request.
