# Supplementary material for: Integrating Multi‐Omics Data Using Machine Learning to Explore New Therapeutic Targets for Acute Kidney Injury
Source: J Cell Mol Med. 2025 Aug 27;29(16):e70801. doi: 10.1111/jcmm.70801 (PMC12385122; doi:10.1111/jcmm.70801)
Supplement: Supplementary file 1 — Appendix S1: jcmm70801‐sup‐0001‐Supinfo.pdf. [file JCMM-29-e70801-s001.pdf]

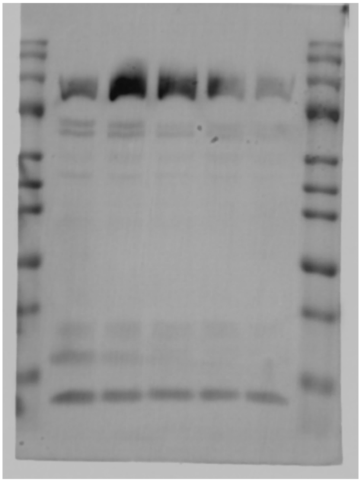

8A.ADAMTS1

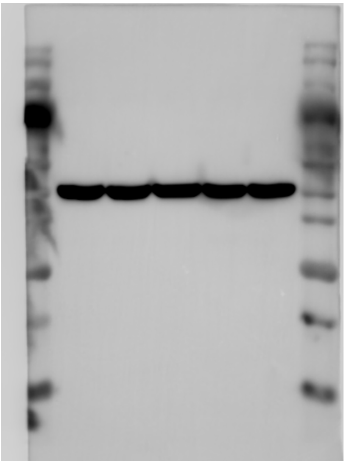

8A.ADMATS1-β-actin

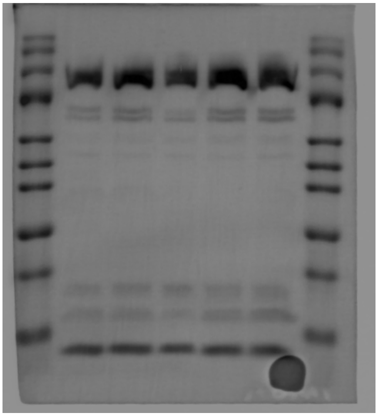

8C.ADAMTS1

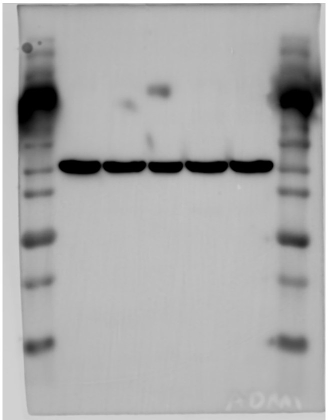

8C. ADAMTS1-β-actin

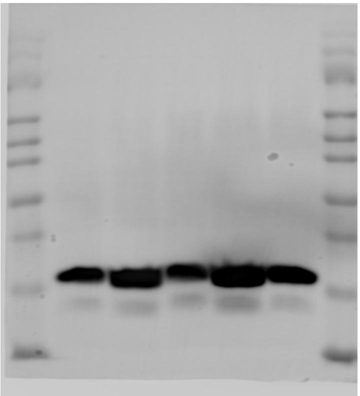

8C.TNFa

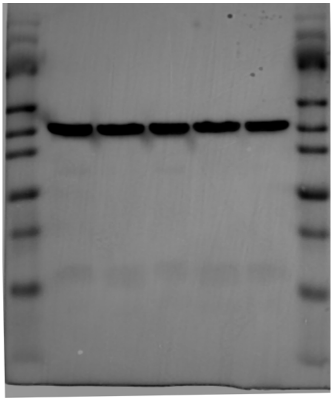

8C.TNFa-β-actin

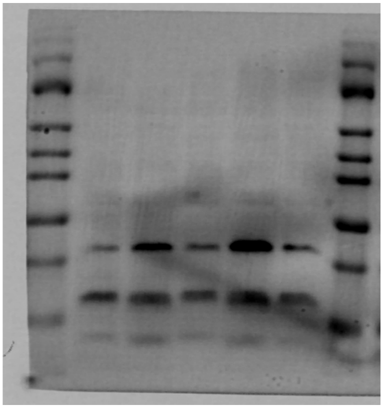

9A.Bax

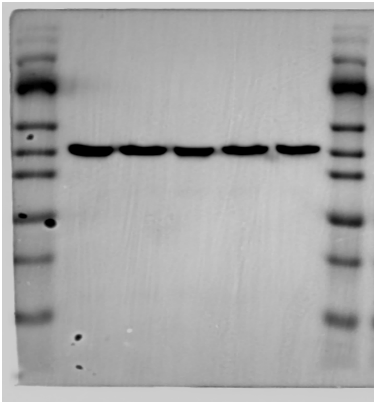

9A.Bax-β-actin

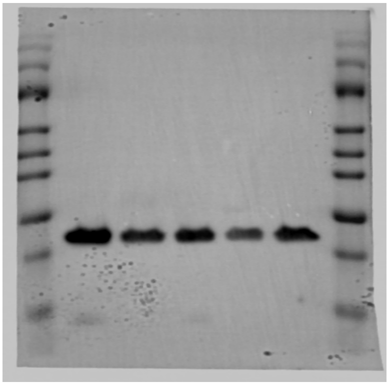

9A.Bcl-2

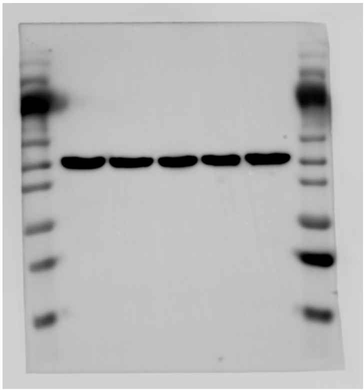

9A.Bcl-2-β-actin

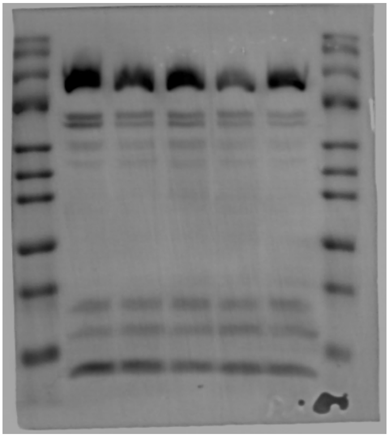

9A.PGC-1α

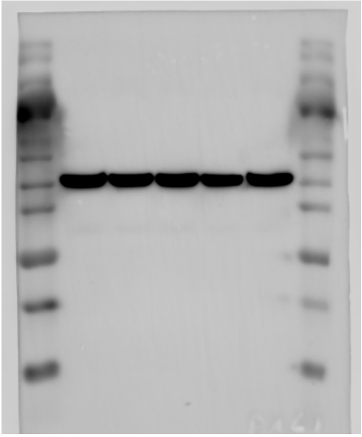

9A.PGC-1α-β-actin

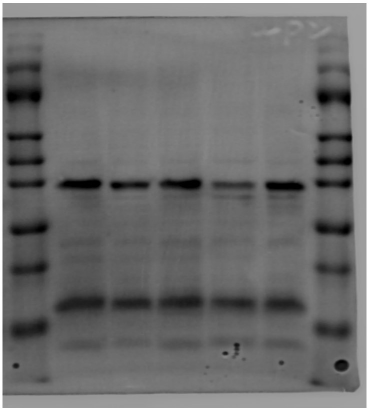

9A.UCP2

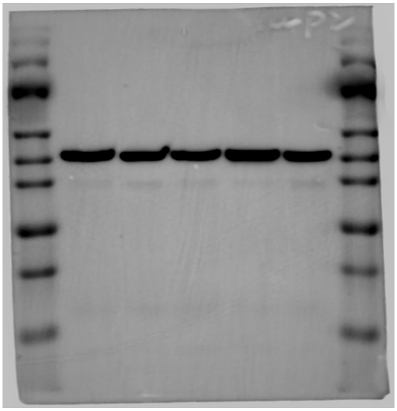

9A.UCP2-β-actin

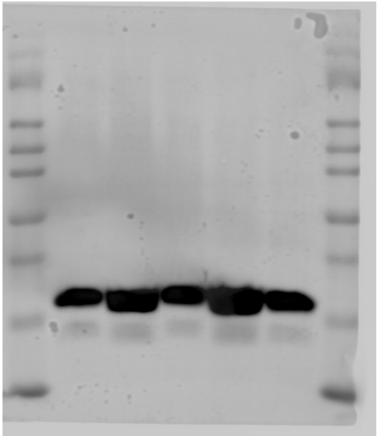

9A.cyt-c

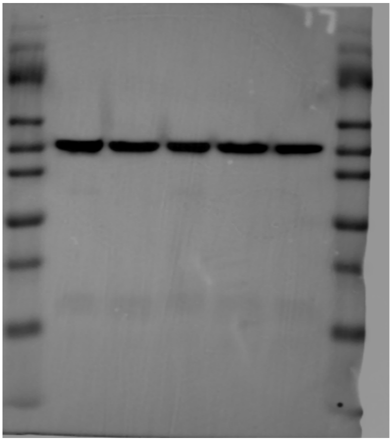

9A.cyt-c-β-actin
